# Efficacy of laparoscopic fundoplication in patients with chronic cough and gastro-oesophageal reflux

**DOI:** 10.1007/s10388-022-00953-2

**Published:** 2022-10-06

**Authors:** Adam Frankel, Hock Soo Ong, B. Mark Smithers, Les K. Nathanson, David C. Gotley

**Affiliations:** 1 Discipline of Surgery, The University of Queensland, Princess Alexandra Hospital Clinical School, Level 4, Building 1, 199 Ipswich Rd, Woolloongabba, QLD 4102 Australia; 2 Department of General Surgery, Singapore General Hospital, Singapore, Singapore; 3 Mater Private Hospital, South Brisbane, QLD Australia; 4 Department of Surgery, Royal Brisbane Hospital, Herston, QLD Australia

**Keywords:** Gastroesophageal reflux, Cough, Laparoscopy, Fundoplication

## Abstract

**Background:**

The outcome of anti-reflux surgery in patients with suspected gastro-oesophageal reflux-induced cough is frequently uncertain. The aims of this study were to assess the efficacy of laparoscopic fundoplication for controlling cough in patients with chronic cough without asthma, who have pathologic gastro-oesophageal reflux, and to identify predictors of response.

**Methods:**

From a prospective database of 1598 patients who have undergone laparoscopic fundoplication, 66 (4%) with proven gastro-oesophageal reflux disease (GORD) and chronic cough without asthma were studied. All patients underwent gastroscopy and 24-h pH monitoring before operation. Heartburn and regurgitation were assessed using a modified DeMeester score. Severity of cough before and after surgery was self-assessed by the patient using a visual analog scale at a minimum of 12 months post-operatively (median 43 mo; range: 14–104 mo). Patients were considered to have responded to fundoplication if they had no cough or the cough had improved by 50% or more after operation.

**Results:**

Cough and heartburn/regurgitation were relieved in 61% (40/66) and 90% (44/49) of the patients, respectively. The presence of typical GORD symptoms or oesophagitis, and pH study variables did not predict the response of the cough to fundoplication.

**Conclusion:**

Refinement in the aetiological diagnosis of chronic cough due to GORD is necessary for improved outcome. Patients diagnosed with GORD-related chronic cough need to be counseled regarding their expectations from anti-reflux surgery.

## Introduction

Gastro-oesophageal reflux disease (GORD) is common, particularly in Western society [1]. The role of laparoscopic fundoplication (LF) in the treatment of patients with typical symptoms of GORD (heartburn and regurgitation) is well established [2, 3]. GORD can also have extra-oesophageal manifestations, including in the larynx and airways [4]. It is estimated that up to 10% of all ENT consultations relate to GORD, with proposed symptoms as diverse as pain (non-descript or burning), throat clearing, globus, chronic cough, hoarseness, nocturnal laryngospasm and cervical dysphagia [5].

Chronic cough has been defined as persistent, troublesome cough for a minimum duration varying from 3 up to 8 weeks [6]. Its adverse effects on the patient’s psychosocial and physical well-being have been previously documented [7]. GORD together with upper airway cough syndrome [UACS, previously known as post-nasal drip syndrome (PNDS)], chronic bronchitis and asthma are the four commonest causes of chronic cough [8]. Asthma itself may be triggered by GORD, creating a complex clinical interaction of cause and effect [9]. In around a quarter of cases, more than one aetiological factor is evident [8].

Unlike the outcome in patients presenting with typical GORD, the efficacy of both medical [10] and surgical [11, 12] treatment in patients primarily with laryngopulmonary symptoms is less clear [13]. A recent systematic review and meta-analysis of LF for the control of respiratory symptoms of GORD found modest evidence of benefit for surgery, but there was considerable heterogeneity in the pre-treatment diagnosis of both GORD and pulmonary conditions, as well as the treatment provided [14]. The included studies were predominantly cohort studies with few comparative studies and no randomised controlled trials. In addition, patients with chronic cough without asthma have been reported to respond differently following anti-reflux surgery compared with patients with asthma [15, 16].

The aims of this study were first to assess the efficacy of LF in patients who present with the predominant symptom of chronic cough and who have objective evidence of GORD. Second, we aimed to identify any pre-operative clinical predictors for a beneficial response to LF.

## Methods

### Work-up

A prospective database captured all patients on whom LF was performed at two tertiary referral centres. This paper refers to results obtained from patients operated on between 1 November 1991 and 31 December 1999. Preoperative investigations included oesophago-gastro-duodenoscopy endoscopy to identify the presence of reflux oesophagitis or Barrett’s oesophagus, 24-h ambulatory dual channel oesophageal pH monitoring and at the consulting surgeon’s discretion, oesophageal manometry, which was generally used when there were symptoms suggestive of oesophageal dysmotility. Oesophageal body peristalsis and lower oesophageal sphincter tone were measured during ten water swallows of 5 ml, 30 s apart. Patients were considered to have oesophageal dysmotility if they had a “non-specific motor disorder”, “low amplitude disorder” or “aperistalsis” [17].

All patients presenting with predominantly laryngopulmonary symptoms had been assessed by thoracic physicians and/or otorhinolaryngology surgeons prior to referral for consideration of anti-reflux surgery. In some patients, a gastroenterologist had also been consulted. All patients were non-smokers or ex-smokers and had the diagnoses of UACS and chronic bronchitis excluded. Asthma was excluded on the basis of a histamine challenge test and/or the absence of broncho-responsiveness to bronchodilators. None were taking cough-inducing medications (e.g., angiotensin converting enzyme inhibitors). Referral for surgery was prompted by failure of medical treatment including proton-pump inhibitors (PPI), with failure defined as lack of satisfactory initial symptomatic response or return of the cough after a period of satisfactory clinical response, or intolerance to medications (due to side effects or non-compliance).

### Surgery

All patients underwent a general anaesthetic and were positioned supine or in lithotomy, at the surgeon’s discretion. If present, a hiatus hernia was repaired (without mesh), ensuring an adequate length of intra-abdominal oesophagus. Most patients underwent a 360° Nissen fundoplication using a standard technique previously described [2]. Selected patients underwent laparoscopic posterior 270° (Toupet) fundoplication or laparoscopic anterior 180° fundoplication [18] if, in the opinion of the operating surgeon, a full wrap was contraindicated because of oesophageal dysmotility detected by pre-operative manometry.

### Follow-up

All patients were enrolled in a standard follow-up program, whereby they were assessed for their symptoms at 1, 3 and 5 years post-operatively by an independent research nurse. For the purposes of this study, the most recent assessment was taken as their current symptom status, except where re-operation had been undertaken, in which case the patient’s pre-revision status was used. Cough was subjectively assessed using a visual analog scale (self-assessment by patients with 0 being no cough and 10 being the worst cough they could imagine) pre-operatively and at least 12 months post-operatively. DeMeester symptom scores [19] were used to assess the severity of heartburn, regurgitation and dysphagia, and were prospectively collected pre-operatively and at 1 and 3 years post-surgery. Each symptom was scored 0–3 in order of increasing severity. Briefly, absence of the symptom scored zero, an occasional non-troublesome symptom scored 1, a score of 2 was allocated for a symptom occurring more than once per week, and 3 was allocated for daily, or nocturnal symptoms requiring long-term PPI therapy. For dysphagia, a score of 2 is allocated for difficulty swallowing requiring liquids to clear two or more times per week, and 3 is for bolus obstruction requiring medical intervention, or the need to avoid certain foods altogether. A score of 2 or 3 is considered to be clinically significant. Patient satisfaction with laparoscopic fundoplication at 1 year was assessed by asking them whether they would go through the operation again given their experience with it.

### Statistics

Cough scores were correlated with pre-operative clinical variables including patient age, weight, gender, presence and severity of reflux symptoms, endoscopic findings and 24-h ambulatory oesophageal pH monitoring, both proximal and distal. Statistical analyses were performed using SPSS for Windows, Release 9.0.0 (SPSS Inc., Chicago, IL, USA). Cross-tabulated comparisons between groups for categorical variables were achieved by *χ*^2^ analysis. The two-tailed t test was used for comparisons of means and Mann–Whitney U test for non-parametric data.

### Ethics

All patients in this study provided informed written consent to have their data included. The study complied in all aspects with the National Health and Medical Research Council of Australia’s Guidelines on Human Research.

## Results

### Clinical characteristics

**Fig. 1:**
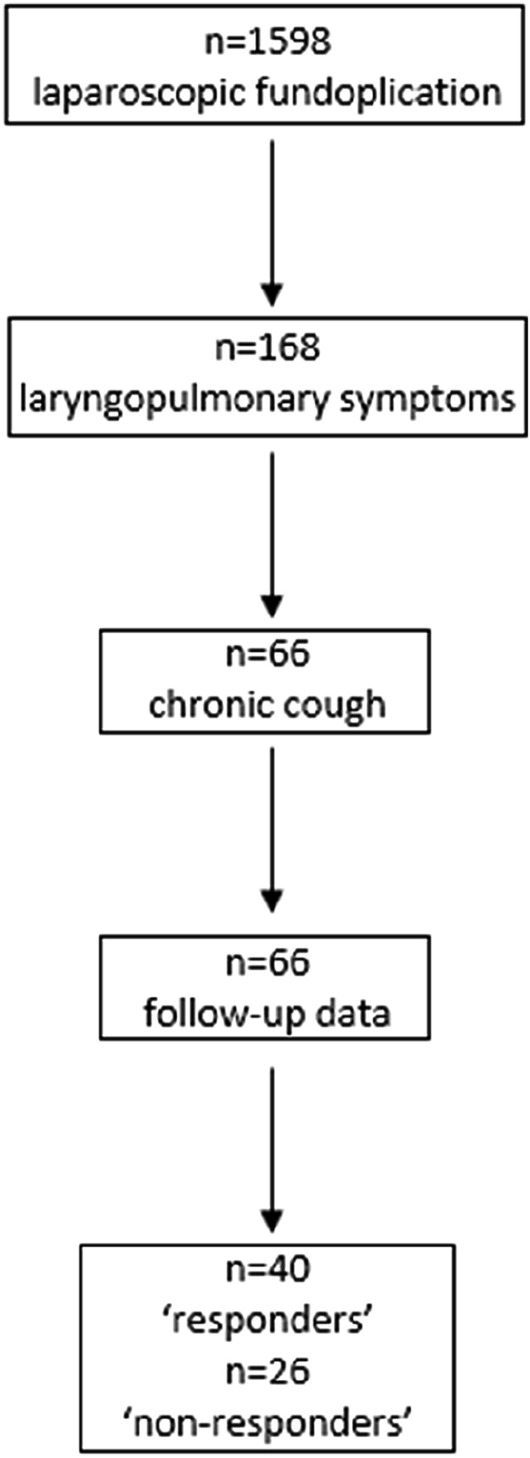
Flow diagram of study population, follow-up and outcomes

Of the 1598 patients in the database with proven GORD undergoing laparoscopic fundoplication, 168 (10.5%) presented with laryngopulmonary symptoms (Fig. 1). The distribution of laryngopulmonary symptoms is shown in Table 1. 66/168 had chronic cough as their principal presenting symptom. The median age of this group was 56 years (31–76 years), 41 patients (62%) were female, and median follow-up was 43 months (12–104 months).

**Table 1:** Frequency of different laryngopulmonary symptoms in 168 patients undergoing laparoscopic fundoplication

Laryngopulmonary symptoms	*n* = 168
Asthma	*n* = 84 (50%)
Chronic cough	*n* = 66 (39%)
Pneumonia/COAD/pulmonary fibrosis	*n* = 12 (7%)
Hoarse voice/vocal cord granuloma/laryngitis	*n* = 6 (4%)

The 66 patients had chronic cough for a median of 48 months (5–240 months) prior to surgical consultation. The median pre-operative cough score was 9.5 (5–10). The other pre-operative clinical features are shown in Table 2. All 66 patients had GORD confirmed on the basis of endoscopic evidence of ulcerative reflux oesophagitis and/or a positive result on 24-h ambulatory pH monitoring (total time pH < 4 > 4% of study time). In 49/66 (74%) patients, cough was associated with clinically significant symptoms of heartburn and/or regurgitation with a DeMeester score of 2 or greater. The remaining 17/66 (26%) did not have any clinically significant heartburn and/or regurgitation (score ≤ 1), but had endoscopic features of reflux oesophagitis and/or a positive pH study.

**Table 2:** Preoperative clinical characteristics of patients with GORD-related chronic cough

Oesophageal manometry	*n* = 66	(%)
LOSP(mmHg)		
0	10	15
1–10	41	62
> 10	13	20
Not measured	2	3
Motility study		
Normal	46	70
NSMD	10	15
Low amplitude	7	11
Aperistaltic	1	2
No studied	2	3
Hiatus Hernia		
Yes	25	38
No	33	50
Unknown	8	12

Endoscopic and pH study findings	*n* = 66	(%)
Oesophagitis		
Nil	25	43
Erythema	9	16
Linear ulcers	15	23
Confluent ulcers	5	9
Barrett’s	1	2
Not available	3	6
24-h distal pH study (pH < 4)		
Total acid exposure ≤ 4%	16	24
Total acid exposure > 4%	41	62
Not available	9	14
24-h proximal pH study (pH < 4)		
Total acid exposure ≤ 0.6%	7	11
Total acid exposure > 0.6%	30	46
Not assessed	29	44

### Operative details

The median operation time was 80 min (40–230 min). Fifty eight (88%) had a laparoscopic Nissen fundoplication, 6 (9%) had a laparoscopic Toupet fundoplication and 2 (3%) had a laparoscopic anterior fundoplication. The superior short gastric vessels were divided in 94% of cases. One procedure was converted to open fundoplication due to equipment failure. Median length of stay was 3 days (1–14 days).

### Morbidity and mortality

Three patients (4.5%) suffered early morbidity (within 30 days of surgery). One was readmitted with acute dysphagia after discharge; this resolved with conservative treatment. Two patients who had undergone laparoscopic Nissen fundoplication suffered immediate severe dysphagia and required early re-operation for conversion to a partial fundoplication. Two patients (3%) experienced late morbidity, both suffering severe chronic dysphagia (DeMeester score 3). They underwent endoscopic dilatation and one subsequently had a conversion from a Nissen to a Toupet fundoplication resulting in resolution of dysphagia. There was no operative mortality in this series.

### Post-operative assessment

Cough was assessed post-operatively in all 66 patients at a median of 43 months (range 12–104 months) and summarised in Table 3. The median post-operative cough score was 3 (0–10). Twenty patients (30%) had no cough (complete resolution) with a further 20 (30%) having an improvement of 50% or greater. Post hoc analysis showed an improvement of 50% or greater correlated most closely with the patient’s satisfaction rating, and hence, these two subgroups were combined (*n* = 40) and subsequently referred to as the “responder” group. Eight patients (12%) had improvement of less than 50%, 16 (24%) had no change and 2 (3%) felt that their cough was worse than before operation. These were combined (*n* = 26) to create the “non-responder” group.

**Table 3:** Summary of cough-related outcomes. Cough was rated using a visual analog scale ranging from 0 (no cough) to 10

	*n*	%
Pre-op	66	100
Post-op	46	70
Improvement		
Complete	20	30
>50%	20	30
<50%	8	12
None	16	24
Worse	2	3

DeMeester symptom scores before and after operation are shown in Table 4. 38/66 (58%) Patients had clinically significant heartburn pre-operatively, which reduced to 2/66 (3%) at 1 year. 43/66 (65%) patients had clinically significant regurgitation pre-operatively, which reduced to 5/66 (8%) patients at 1 year, Combining these two metrics, 60/66 (91%) patients were free of any clinically significant heartburn or regurgitation at 1 year. At 1 year, 67% of patients were satisfied with the results of their surgery, 20% were not and 13% were unsure. For comparison, patients without laryngopharyngeal symptoms but with typical heartburn and regurgitation undergoing laparoscopic fundoplication during the same study period had a satisfaction rate at 1 year of 94%. Data were available for 37 patients at 3 years. Clinically significant heartburn increased to 3/37 (9%) at 3 years, although no patients had a symptom score of 3, with clinically significant regurgitation in 1/37 (3%) at 3 years. Dysphagia marginally increased from 10/66 (15%) pre-operatively to 13/66 (20%) at 1 year, but dropped to 1/37 (3%) by 3 years.

**Table 4:** Summary of symptoms pre-operatively and at 1 and 3 years after operation in 66 patients having anti-reflux surgery for chronic cough

DeMeester Symptom Score		0	1	2	3	Total
Heartburn	Pre-op	17 (26%)	11 (16%)	17 (26%)	21 (32%)	66
	1 year	47 (71%)	17 (26%)	0	2 (3%)	66
	3 years	26 (70%)	8 (21%)	3 (9%)	0	37
Regurgitation	Pre-op	12 (18%)	11 (17%)	23 (35%)	20 (31%)	66
	1 year	50 (76%)	11 (17%)	3 (4%)	2 (3%)	66
	3 years	30 (81%)	6 (16%)	0	1(3%)	37
Dysphagia	Pre-op	40 (61%)	16 (24%)	10 (15%)	0	66
	1 year	36 (54%)	17 (26%)	11 (17%)	2 (3%)	66
	3 years	26 (70%)	10 (27%)	1(3%)	0	37

### Predictors of response

Clinical variables were compared between the responders and non-responders. No significant predicting factors were identified (Table 5), although there was a trend towards response for younger patients, higher weight and those with oesophageal dysmotility doing better. In particular, severity of reflux in terms of symptom score, degree of oesophagitis or extent of acid exposure either in the proximal or distal oesophagus, were not significantly different between responders and non-responders. Other variables derived from 24-h pH monitoring including nocturnal and diurnal mean acid exposure time; total, nocturnal and diurnal number of reflux episodes, number of reflux episodes > 5 min and duration of longest reflux episode, and correlation between acid reflux events and cough events were also not different between responders and non-responders (data not shown).

**Table 5:** Comparison of clinical characteristics between responders and non-responders to anti-reflux surgery. Comparisons statistically analysed using Chi-squared test

	Responders (*n* = 40)	Non-responders (*n* = 26)	*P* value
Mean age (years) (SD)	52.8 (11.0)	58.7 (11.3)	0.051
Mean weight (kg) (SD)	79.4 (12.0)	73.1 (10.6)	0.054
Median duration of symptoms (months) (range)	48 (5–240)	60 (12–240)	0.260
% With heartburn (DeMeester score 2,3)	58	48	0.429
% With regurgitation (DeMeester score 2,3)	64	57	0.571
% With hiatus hernia	43	43	0.973
% With oesophagitis	58	55	0.807
% With oesophageal dysmotility	77	55	0.074
Mean total acid (pH < 4) exposure time at proximal probe, *n* = 33 (SD)	4.7% (7.1)	5.7% (9.5)	0.727
Mean total acid (pH < 4) exposure time at distal probe, *n* = 50 (SD)	9.7% (8.1)	9.7% (8.1)	0.999

## Discussion

Chronic cough of unknown aetiology is a common clinical problem. The prolonged duration of cough (median 48 months) prior to surgical referral for LF seen in this cohort reflects the difficulty in diagnosis and effective treatment of many patients with chronic cough. Those referred for consideration for LF will usually have had asthma, UACS and pulmonary parenchymal disease ruled out as aetiological factors. Chronic cough and GORD are both common, making it difficult to establish gastro-oesophageal reflux as a causative factor of chronic cough. Clinical decision-making is further complicated by the often complex and multifactorial aetiology of chronic cough [8].

Twenty-four hour ambulatory pH monitoring has long been proposed as an effective investigation for the diagnosis of GORD-related cough [20]. Further refinement of this investigation with the addition of a proximal pH electrode was considered an advance [21]; however, the degree of proximal acidification may not be more discerning in detecting those with reflux-related laryngopulmonary symptoms than distal acidification alone [22]. Activation of vagal afferent C-fibres induces cough-reflex hypersensitivity [23]. While there are no convincing human data that such activation can trigger the cough reflex per se [24], there are animal studies suggesting acidification of the oesophagus does cause neurogenic inflammation of the airway, adding further complexity to the relationship between GORD and chronic cough [25].

Another widely heralded advance was the development of symptom association scores, which are now considered an integral part of the investigation of GORD [1]. However, their role in determining whether GORD is responsible for laryngopharyngeal symptoms is still evolving [26]. A combination of oesophageal physiology metrics such as acid exposure time and symptom association score may be the best way to predict response of cough to proton-pump inhibitor [27, 28], which presumably would extrapolate to the efficacy of anti-reflux surgery.

Our cohort represents a select group of patients in whom cough was the sole laryngopulmonary symptom and GORD was demonstrated to be present. Such patients are often referred for consideration for LF as a last resort, and there may be the expectation that control of reflux will lead to elimination of the cough. The cohort has had semi-quantitative grading of both reflux symptoms and cough before and at least 1 year after fundoplication. Follow-up oesophageal pH monitoring was not routinely done, since the primary focus of this study was the patient-reported outcome measure of cough score.

Seventy-four percent of our patients had associated typical symptoms of GORD, with the rest having chronic cough as the sole symptom. However, the presence of typical GORD symptoms did not appear to predict the outcome for chronic cough after LF. As might be expected, LF controlled typical GORD symptoms in > 90% of our cohort. However, only 61% of those with chronic cough experienced significant (> 50%) relief of their cough. The lower response rates seen with chronic cough compared to those with typical GORD symptoms may be attributed to the difficulty in arriving at a complete and accurate aetiological diagnosis of chronic cough. Chronic cough is known to be due to multiple disorders in 26% of patients [8]. Hence, treating GORD alone will not be expected to result in complete relief in these patients. Moreover, some causes of cough are related to, or may trigger, other causes of cough. Chronic cough from any cause may also precipitate GORD in a self-perpetuating cough-reflux cycle [29]. Hence, a positive pH study for GORD in a patient with chronic cough may be precipitated by cough from an alternative aetiology.

Our data contrast with other studies that have reported that the presence of typical GORD symptoms was predictive of cough improvement. For example, Chen and colleagues showed in a cohort of comparable size to ours that 65% of patients with typical GORD symptoms had cough resolution, compared with only 27% of those with atypical symptoms [30]. Falk and colleagues showed improvement in symptom scores for patients with mixed typical GORD and LPR symptoms versus those with LPR symptoms alone, although the degree of improvement was greater in the former [31]. This may be explained by the mixed subgroup having a greater number of symptoms from which they could be saved by LF. Patient satisfaction was > 85% in both subgroups, which is greater than ours (67%) and the cohort from Park et al. (71%) [32]. Aiolfi and colleagues reported improved LPR scores of 91% in the 29 patients with 3-year follow-up, which would presumably correspond with patient satisfaction [33]. A recent systematic review examining improvement in LPR (as measured by the Reflux Symptom Index pre- and post-LF) contained data from nine studies with a total of 287 patients undergoing LF [34]. Only one of these studies reported whether the patient was happy with their operation (Iqbal et al., 85% satisfaction) [35]. Notably, studies in this systematic review had the broad inclusion criteria of symptoms of LPR, rather than chronic cough as the principal symptom.

In our cohort of patients with the principal symptom of cough and objective evidence of GORD, the severity (as measured by standard pH study metrics such as acid exposure time) did not provide any prognostic value. This is in keeping with other similarly designed and sized studies that have found no association between post-fundoplication improvement in LPR symptoms and any metrics from the pH study, including symptom association probability [26, 31]. The ability to predict response to LF, therefore, remains difficult.

Indeed, based on data derived from our current study as well as the published literature, there are no robust pre-operative clinical variables that can be used to predict post-operative outcome in this group of patients. None of the measured variables were statistically predictive of response in our cohort, although there appeared to be a trend to response for those who were younger, higher weight or with oesophageal dysmotility. It is unclear why age may influence response of cough to LF. It seems possible that older patients may have a broader list of possible causes for chronic cough. Other literatures suggests that older patients are more likely to have atypical symptoms and a higher rate of oesophageal dysmotility [36]. This does not necessarily conflict with our data showing response is more likely in young patients or those with oesophageal dysmotility, because these characteristics are not mutually exclusive. Meta-analyses have clearly shown that obesity is a risk factor for GORD, with the prevalence of clinical and endoscopic features increasing with weight [37, 38]. Oesophageal dysmotility has been purported to be a significant contributing factor in chronic GORD-related cough [39]. One possible mechanism is the reduced clearance of refluxed gastric contents [40]. Dysmotility is not reliably corrected by anti-reflux surgery [41], but LF would reduce the volume of oesophageal contents that could otherwise be propelled in an oral direction predisposing the patient to continued aspiration. However, the presence of dysmotility remains of limited to no usefulness in pre-operative selection of patients with cough for LF, just as the presence of dysmotility remains of uncertain significance to surgical outcomes [42].

We have not evaluated response rates for cough in relation to the effectiveness of previous PPI treatment, since most patients in this series had failed medical treatment. All patients referred for operation in this series had already been treated with maximum recommended doses of a PPI (albeit different treatment regimens reflecting the heterogeneous nature of the specialist referral base). One previous report has suggested that patients who respond to PPI therapy do better after anti-reflux surgery than those whose cough has not responded. However, other reports have refuted these findings [43]. PPIs are effective in reducing gastric acidity but may not correct the problem of regurgitation of non-acid material, which may persist in irritating the oesophagus and pharynx and stimulate the cough reflex.

There are several limitations to our study, aside from the inherent problems of a retrospective analysis. We did not have symptom association scores for the cohort to determine if they were predictive of outcome. However, as described earlier, their utility was limited in other similar studies. We also did not have any impedance study information.

Overall, based on our study and available literature, it appears there are no reliable pre-operative clinical predictors for a satisfactory response of cough to LF. This probably reflects an inability to currently obtain a complete and accurate aetiological diagnosis of chronic cough, even in the presence of known abnormal gastro-oesophageal reflux disease. The morbidity from LF in expert centres is low. Therefore, in a well-chosen cohort of patients with chronic cough, LF can yield good patient satisfaction rates, albeit less than one sees in patients who have had the surgery for typical GORD symptoms.

## References

[1] Gyawali CP, Kahrilas PJ, Savarino E (2018). Modern diagnosis of GERD: the Lyon Consensus. Gut.

[2] Gotley D, Smithers B, Rhodes M (1996). Laparoscopic Nissen fundoplication–200 consecutive cases. Gut.

[3] Peters JH, DeMeester TR, Crookes P (1998). The treatment of gastroesophageal reflux disease with laparoscopic Nissen fundoplication: prospective evaluation of 100 patients with" typical" symptoms. Ann Surg.

[4] Nowak K, Sharma S, Kini S, Grams J, Perry KA, Tavakkoli A (2019). Laryngopharyngeal reflux (LPR). The SAGES manual of foregut surgery.

[5] Vaezi MF, Hicks DM, Abelson TI (2003). Laryngeal signs and symptoms and gastroesophageal reflux disease (GERD): a critical assessment of cause and effect association. Clin Gastroenterol Hepatol.

[6] Irwin RS, Boulet L-P, Cloutier MM (1998). Managing cough as a defense mechanism and as a symptom: a consensus panel report of the American College of Chest Physicians. Chest.

[7] French CL, Irwin RS, Curley FJ (1998). Impact of chronic cough on quality of life. Arch Intern Med.

[8] Irwin RS, Richter JE (2000). Gastroesophageal reflux and chronic cough. Am J Gastroenterol.

[9] Irwin RS, Curley FJ, French CL (1993). Difficult-to-control asthma: contributing factors and outcome of a systematic management protocol. Chest.

[10] Sontag SJ, O'Connell S, Khandelwal S (2003). Asthmatics with gastroesophageal reflux: long term results of a randomized trial of medical and surgical antireflux therapies. Am J Gastroenterol.

[11] Swoger J, Ponsky J, Hicks DM (2006). Surgical fundoplication in laryngopharyngeal reflux unresponsive to aggressive acid suppression: a controlled study. Clin Gastroenterol Hepatol.

[12] Sidwa F, Moore AL, Alligood E (2017). Surgical treatment of extraesophageal manifestations of gastroesophageal reflux disease. World J Surg.

[13] Ghisa M, Della Coletta M, Barbuscio I (2019). Updates in the field of non-esophageal gastroesophageal reflux disorder. Exp Rev Gastroenterol Hepatol.

[14] Tustumi F, Bernardo WM, Mariano da Rocha JR (2021). Anti-reflux surgery for controlling respiratory symptoms of gastro-esophageal reflux disease: A systematic review and meta-analysis. Asian J Surg.

[15] Ruth M, Bake B, Sandberg N (1994). Pulmonary function in gastro-esophageal reflux disease-effect of reflux control by fundoplication. Dis Esophagus.

[16] Ekström T, Johansson K-E (2000). Effects of anti-reflux surgery on chronic cough and asthma in patients with gastro-oesophageal reflux disease. Respir Med.

[17] Bessell J, Finch R, Gotley D (2000). Chronic dysphagia following laparoscopic fundoplication. J Br Surg.

[18] Gotley DC, Frankel AJ (2021). Partial Fundoplications (270° Toupet, 90° Dor). Foregut.

[19] Johnson LF, Demeester TR (1974). Twenty-four-hour pH monitoring of the distal esophagus. Am J Gastroenterol.

[20] DeMeester TR, Bonavina L, Iascone C (1990). Chronic respiratory symptoms and occult gastroesophageal reflux. A prospective clinical study and results of surgical therapy. Ann Surg.

[21] Schnatz PF, Castell JA, Castell DO (1996). Pulmonary symptoms associated with gastroesophageal reflux: use of ambulatory pH monitoring to diagnose and to direct therapy. Am J Gastroenterol.

[22] Wo JM, Hunter JG, Waring JP (1997). Dual-channel ambulatory esophageal pH monitoring (a useful diagnostic tool?). Digest Dis Sci.

[23] Javorkova N, Varechova S, Pecova R (2008). Acidification of the oesophagus acutely increases the cough sensitivity in patients with gastro-oesophageal reflux and chronic cough. Neurogastroenterol Motil.

[24] Canning BJ (2010). Afferent nerves regulating the cough reflex: mechanisms and mediators of cough in disease. Otolaryngol Clin N Am.

[25] Chen Z, Sun L, Chen H (2018). Dorsal vagal complex modulates neurogenic airway inflammation in a guinea pig model with esophageal perfusion of HCl. Front Physiol.

[26] Díaz Vico T, Elli EF (2020). Clinical outcomes of gastroesophageal reflux disease-related chronic cough following antireflux fundoplication. Esophagus.

[27] Li N, Chen Q, Wen S (2021). Diagnostic accuracy of multichannel intraluminal impedance-pH monitoring for gastroesophageal reflux-induced chronic cough. Chronic Respir Dis.

[28] Ribolsi M, Lucaguarino MP, Balestrieri P (2021). The results from up-front esophageal testing predict proton pump inhibitor response in patients with chronic cough. Am J Gastroenterol.

[29] Breslin A (1997). The patient with chronic cough. Med J Aust.

[30] Chen D, Wang Z, Hu Z (2019). Typical symptoms and not positive reflux-cough correlation predict cure of gastroesophageal reflux disease related chronic cough after laparoscopic fundoplication: a retrospective study. BMC Gastroenterol.

[31] Falk GL, Gooley SC, Church NG (2020). How effective is the control of laryngopharyngeal reflux symptoms by fundoplication? Symptom score analysis. Eur Surg.

[32] Park A, Weltz AS, Sanford Z (2019). Laparoscopic antireflux surgery (LARS) is highly effective in the treatment of select patients with chronic cough. Surgery.

[33] Aiolfi A, Cavalli M, Saino G (2020). Laparoscopic toupet fundoplication for the treatment of laryngopharyngeal reflux: results at medium-term follow-up. World J Surg.

[34] Morice D, Elhassan H, Myint-Wilks L (2022). Laryngopharyngeal reflux: is laparoscopic fundoplication an effective treatment?. Ann R Coll Surg Engl.

[35] Iqbal M, Batch AJ, Spychal RT (2008). Outcome of surgical fundoplication for extraesophageal (atypical) manifestations of gastroesophageal reflux disease in adults: a systematic review. J Laparoendosc Adv Surg Tech.

[36] Pizza F, Rossetti G, Limongelli P (2007). Influence of age on outcome of total laparoscopic fundoplication for gastroesophageal reflux disease. World J Gastroenterol.

[37] Corley DA, Kubo A (2006). Body mass index and gastroesophageal reflux disease: a systematic review and meta-analysis. Am J Gastroenterol.

[38] Hampel H, Abraham NS, El-Serag HB (2005). Meta-analysis: obesity and the risk for gastroesophageal reflux disease and its complications. Ann Intern Med.

[39] Johnson WE, Hagen JA, DeMeester TR (1996). Outcome of respiratory symptoms after antireflux surgery on patients with gastroesophageal reflux disease. Arch Surg.

[40] Ribolsi M, de Carlo G, Balestrieri P (2020). Understanding the relationship between esophageal motor disorders and reflux disease. Exp Rev Gastroenterol Hepatol.

[41] Fibbe C, Layer P, Keller J (2001). Esophageal motility in reflux disease before and after fundoplication: a prospective, randomized, clinical, and manometric study. Gastroenterology.

[42] Kohn GP (2021). The relevance of ineffective esophageal motility to surgical practice. Foregut.

[43] Wetscher GJ, Glaser K, Hinder RA (1997). Respiratory symptoms in patients with gastroesophageal reflux disease following medical therapy and following antireflux surgery. Am J Surg.

